# CLINICAL MUSCULAR EVALUATION IN PATELLOFEMORAL PAIN SYNDROME

**DOI:** 10.1590/1413-785220182602187215

**Published:** 2018

**Authors:** PAULO LOBO, ITAMAR ALVES BARBOSA, JOSÉ HUMBERTO DE SOUZA BORGES, RODRIGO FERREIRA TOBIAS, MARCOS VINÍCIUS DA SILVA BOITRAGO, MÁRCIO DE PAULA OLIVEIRA

**Affiliations:** 1. IPE-HOME, Hospital Ortopédico e Medicina Especializada, Brazilia, DF, Brazil.

**Keywords:** Patellofemoral pain syndrome/physiopathology, Patellofemoral pain syndrome/therapy, Knee joint, Muscle, skeletal/physiology., Síndrome da dor patelofemoral/fisiopatologia, Síndrome da dor patelofemoral/terapia, Articulação do joelho, Músculo esquelético/fisiologia.

## Abstract

**Objective:**

To define a profile of the muscle groups affected by patellofemoral pain syndrome (PFPS) to determine a pattern of functional weaknesses around the knee.

**Methods:**

Sixty-three female patients were randomly selected, and 17 included in this study, receiving a clinical evaluation with pre-established protocol which evaluated the quadriceps, abductors, range of motion in the internal rotators and ankle dorsiflexors, pelvic tilt, and dynamic valgus.

**Results:**

Losses were seen in abductor strength and ankle dorsiflexor range of motion in comparison with the contralateral limb (0.031 and 0.040, respectively). There was a loss of quadriceps strength and decreased amplitude of internal hip rotation when compared to the Kujala score (0.032 and 0.002, respectively). Dynamic valgus and pelvic fall were greater in comparison with the Kujala score (0.668 and 0.567, respectively).

**Conclusion:**

Clinical evaluation showed deficits in the quadriceps and abductor muscle groups, as well as decreased range of motion in the internal hip rotators and ankle dorsiflexors and increased dynamic valgus and pelvic drop. Level of Evidence IV; Case series.

## RESUMO

**Objetivo:**

Definir o perfil de acometimento de grupos musculares envolvidos na síndrome de dor patelofemoral (SDPF) a fim de determinar um padrão de fraqueza funcional em torno do joelho.

**Métodos:**

Foram selecionados randomicamente 63 pacientes do sexo feminino, dos quais 17 foram incluídos no trabalho e submetidos a uma avaliação clínica com protocolo preestabelecido que avaliou quadríceps, abdutores, amplitude de movimento de rotadores internos e dorsiflexores de tornozelo, além de inclinação pélvica e valgo dinâmico.

**Resultados:**

Houve déficit de força de abdutores e diminuição de amplitude do movimento de dorsiflexores de tornozelo em comparação com o membro contralateral (0,031; 0,040, respectivamente). Houve déficit de força de quadríceps e diminuição de amplitude de rotação interna do quadril quando em comparação com o escore de Kujala (0,032; 0,002, respectivamente). Verificou-se aumento do valgo dinâmico e queda pélvica com relação ao escore de Kujala (0,668; 0,567, respectivamente).

**Conclusões:**

Após avaliação clínica dos pacientes incluídos, observaram-se déficits dos seguintes grupos musculares: quadríceps e abdutores, além da diminuição da amplitude de movimento de rotadores internos do quadril e dorsiflexores de tornozelo; por fim, foram identificados aumento do valgo dinâmico e queda pélvica. Nível de Evidência IV; Série de casos.

## INTRODUCTION

The yearly incidence of anterior knee pain reaches 22 out of every 1000 randomly selected people,^1^ ^,^ ^2^ approximately 17 of whom are young women.^1^ ^-^ ^3^ Patellofemoral pain syndrome (PFPS) has a broadly discussed terminology due to the variety of symptoms it presents; this condition is also known as runner’s knee, patellar pain, patellar chondropathy, patellar overload syndrome, and patellofemoral pain.^4^ ^,^ ^5^ Furthermore, PFPS is a diagnosis of exclusion, after discarding other pathologies that may cause anterior knee pain, such as degenerative, structural, mechanical or neurological pathologies.^5^ ^,^ ^6^

The etiology of PFPS is multifactorial; notable components include impairment of neuromuscular control of the trunk, pelvis, and legs during functional activities, particularly with regard to the imbalance of forces involved in the musculature around the knee.^7^ Moller et al.^8^ found an unbalance between the quadriceps and the hamstrings in patients with PFPS compared with a control group when assessing muscle activities via electroneuromyography.

Currently, neuromuscular training has been gaining prominence as an excellent technique for use in clinical practice, but there is no consensus in the literature as to a specific method for neuromuscular training or even until what point neuromuscular imbalance can lead to anterior knee pain. Consequently, many authors have emphasized exercises to strengthen the vastus medialis obliquus (VMO).^9^ But Cerny^10^ reported that exercises strengthening the vastus lateralis were as important as those strengthening the VMO.

The objective of this study is to define a profile of muscular involvement and mechanical changes involved in PFPS in order to determine a pattern of involvement for functional weaknesses around the knee, optimizing future training protocols to treat PFPS.

## MATERIALS AND METHODS

We randomly selected 63 female patients; of these, 17 with knee pain were effectively assessed after the exclusion criteria were carefully applied. All patients underwent magnetic resonance imaging (MRI) and were evaluated by the same team composed of three orthopedic physicians and two physiotherapists.

The inclusion criteria were: female patients between eighteen and forty-five years with anterior knee pain. The exclusion criteria were: any degenerative changes of the knee or hip, history of patellar dislocation, clinical evidence of knee instability, meniscal or other intra-articular injuries, evidence of edema, Osgood-Schlatter disease or Sinding-Larsen-Johanssen syndrome, patellar tendinopathy, chondral injury, osteoarthritis, neurological involvement affecting the gait, joint or muscle injuries in the hip, lumbar pain, sacroiliac pain, patients who previously received surgical treatment in the knee or hip joints, and bilateral complaint of anterior knee pain, since all patients were assessed in comparison with the healthy knee.

Within the sample group, muscular strength was assessed in the abductors (SA), in the external rotators of the hip (SEHR), and the quadriceps (QS). A calibrated Hand Held MicroFET 2 manual dynamometer (Hoggan Scientific, Salt Lake City, USA) was used to assess these parameters. Range of motion was assessed via ankle dorsiflexion (RAD) and internal rotation of the hip (RIHR). A calibrated AM-2 angle meter (Starrett, Athol, MA, USA) was also used.

The EVA and Kujala scores were calculated, in addition to the degree of dynamic valgus and pelvic drop.

Each calculation was repeated three times and the average of these values was used.

The results were statistically evaluated by the most appropriate method, using SPSS software (IBM, Armonk, NY, USA). All tests were performed at a 5% significance level.

This study was approved by the Brasil Platform for ethics in human research (CAAE: 60238116.9.0000.0023) and an informed consent form was signed by all patients.

## RESULTS

Clinical evaluation of the groupings in which muscular strength was measured showed only a loss of strength in comparison with the contralateral knee, with a statistically significant result (0.031) for the abductors. (Table 1)

**Table 1:** Difference between mean values for lower limbs.

Difference between mean values for lower limbs.			
	Contralateral side	Affected side	Sig***
RAD*	41.17	39.83	0.040
SA**	23.26	21.03	0.031

The level of significance is 0.05, according to the Wilcoxon test for related samples

When range of movement was assessed, loss was seen in RAD (0.040), as demonstrated in Table 1.

Comparison of dynamic valgus and pelvic drop correlated to the Kujala score (0-100; the lower the score, the greater the weakness of the knee) showed statistically significant results (0.668 and 0.567, respectively). (Table 2) According to the B coefficient (linear regression coefficient) for each grade plus the difference between the mean averages for the legs in dynamic valgus or pelvic drop, there was a decrease of 1.502 and 2.544 times that of the Kujala score, respectively. (Table 2)

**Table 2:** Relation between difference in the means between lower limbs and Kujala score.

Relation between difference in the means between lower limbs and Kujala score.		
	B*	Sig ****
Pelvic Drop	-2.544	0.010
Dynamic Valgus	*-1502	0.003
QS**	0.714	0.032
RIHR***	1.133	0.002

*= relationship is inversely proportional between Kujala score and variable; **= quadriceps strength; ***= Amplitude of internal hip rotators; ****= significance.

There was a loss in QS and decrease in RIHR when these variables were correlated to the Kujala score (0.032 and 0.002, respectively). (Table 2) According to the B coefficient, for each degree reduced between the mean difference for each leg in RIHR or for each Newton of force reduced in the difference between the mean QS values, there was a 1.133 and 0.714-fold reduction in the Kujala score, respectively.

## DISCUSSION

PFPS is known to involve anterior knee pain without any chondral changes in the femoropatellar joint, according to Petersen et al.^11^ Muscle imbalance is believed to be one of the main factors that increases the risk of PFPS.^4^ ^,^ ^12^ ^-^ ^15^ This fact is confirmed by the results of this study, which found a significant reduction in SA and QS.

Recent studies have shown that PFPS does not appear in the knee joint, but rather in the decreased amplitude of internal rotation of the femur, due to weakness of the hip abductors (gluteus medius and minimus muscles).^16^ ^-^ ^20^ Both of these results were statistically determined in this study.

Weakness of the gluteus medius and minimus muscles causes pelvic instability, and consequently the patient cannot support the pelvis for one minute while standing on the affected leg, thus determining pelvic drop, as reported by Petersen et al.^11^ Moreover, weakness in these muscles lead to internal rotation of the femur, thus decreasing RIHR. These statements were confirmed by the assessment of pelvic angle in this study.

Besides pelvic instability, weakness of the hip muscles causes a leg alignment known as dynamic valgus.^21^ ^,^ ^22^ This biomechanical and muscular mechanism may be strongly linked to the pathogenesis of PFPS.^11^ ^,^ ^23^ ^,^ ^24^ This assessment pattern (dynamic valgus) was seen to have a strong influence on the pathogenesis of PFPS in our results (0.003).

To the best of our knowledge, no single assessment combining results related to the muscular forces around the knee and the biomechanical changes that corroborate with the etiology of PFPS can be found in the recent literature. Although we effectively evaluated 17 patients, a number that could represent bias in our results, this bias was minimized by the regularity and expressive statistical results of the evaluated parameters, and our study was strengthened by the selective exclusion method applied.

Understanding PFPS is absolutely essential in order to be able to treat this condition, and our results can guide future training efforts for treatment.

## CONCLUSIONS

Patients with PFPS in this study demonstrated a reduction in SA, decrease in RIHR and reduction of RAD, and increase in pelvic tilt and dynamic valgus.
